# Single neonatal dexamethasone administration has long-lasting outcome on depressive-like behaviour, *Bdnf, Nt-3, p75ngfr* and sorting receptors (*SorCS1-3*) stress reactive expression

**DOI:** 10.1038/s41598-021-87652-7

**Published:** 2021-04-14

**Authors:** D. A. Lanshakov, E. V. Sukhareva, V. V. Bulygina, A. V. Bannova, E. V. Shaburova, T. S. Kalinina

**Affiliations:** 1 Laboratory of Postgenomics Neurobiology, Institute of Cytology and Genetics, Russian Academy of Science, Novosibirsk, Russian Federation 630090; 2 Functional Neurogenomics Laboratory, Institute of Cytology and Genetics, Russian Academy of Science, Novosibirsk, Russian Federation 630090; 3 Department of Natural Sciences, Novosibirsk State University, Novosibirsk, Russian Federation 630090

**Keywords:** Cellular neuroscience, Development of the nervous system, Molecular neuroscience, Neurotrophic factors, Stress and resilience

## Abstract

Elevated glucocorticoid level in the early postnatal period is associated with glucocorticoid therapy prescribed at preterm delivery most often has severe long-lasting neurodevelopmental and behavioural effects. Detailed molecular mechanisms of such programming action of antenatal glucocorticoids on behaviour are still poorly understood. To address this question we studied neurotrophins: *Bdnf*, *Nt-3*, *Ngf* and their receptors: *p75ngfr*, *Sorcs3* expression changes after subcutaneous dexamethasone (DEX) 0.2 mg/kg injection to P2 rat pups. Neurotrophins expression level was studied in the hippocampus (HPC). Disturbances in these brain regions have been implicated in the emergence of multiple psychopathologies. *p75ngfr* and *Sorcs3* expression was studied in the brainstem—region where monoamine neurons are located. Immunohistochemically P75NTR protein level changes after DEX were investigated in the brainstem Locus Coereleus norepinephrine neurons (NE). In the first hours after DEX administration elevation of neurotrophins expression in HPC and decline of receptor’s expression in the NE brainstem neurons were observed. Another critical time point during maturation is adolescence. Impact of elevated glucocorticoid level in the neonatal period and unpredictable stress (CMUS) at the end of adolescence on depressive-like behaviour was studied. Single neonatal DEX injection leads to decrease in depressive-like behaviour, observed in FST, independently from chronic stress. Neonatal DEX administration decreased *Ntf3* and *SorCS1* expression in the brainstem. Also *Bdnf* mRNA level in the brainstem of these animals didn’t decrease after FST. CMUS at the end of adolescence changed *p75ngfr* and *SorCS3* expression in the brainstem in the animals that received single neonatal DEX administration.

## Introduction

Early period of life is crucial for the proper organism development especially the brain and nervous system^1–3^. Interventions at these vulnerable periods could be fatal for the proper maturation, normal behaviour and mental abilities^4–7^. Factors that influence early development are numerous, but most often seen in ordinary life are elevated glucocorticoid’s level relative to the mother’s stress, or glucocorticoid therapy aimed to prevent respiratory distress syndrome^8–10^. Glucocorticoids (GC) regulate diverse physiological functions and are essential for embryo development, metabolism, lung maturation and survival^11–13^. They play a key role in stress response and HPA axis regulation. GCs perform their function via glucocorticoid receptor (GR)—ligand activated transcription factor^14–16^. Perinatal GC exposure causes developmental and behaviour abnormalities and has severe negative influence on further development of the nervous system^17–19^. In the neonatal brain GC causes neurons and progenitor’s apoptosis^20,21^. Glucocorticoids could induce neuron apoptosis in the neonatal brain via glutamatergic excitatory mechanism in the subicular region^4^ and shapes further neural circuits and animal’s behaviour. Hippocampal function is particularly influenced by glucocorticoids, because GR is highly expressed in this brain structure^22^. In animals that come through maternal separation stress hippocampus-dependent behaviour is affected. Spatial and working memory, reaction to novelty are alternated by acute modulation of neuronal excitability^23,24^ and direct changing of GR dependent gene expression. Glucocorticoids could affect brains and cognition also with stress during adolescence. It can disrupt developmental trajectories controlling reproduction, cognition, and the ability to respond to adversity typically what leads to phenotypic alterations in adulthood. Remarkably, in animal experiments different types of stressors during adolescence (predictable, unpredictable and restraint stress) had different outcomes on stress-resilience and memory extinction in rats through *Bdnf* signaling in the infralimbic cortex^25^.

Nerve growth factors and their receptors are key players in shaping further neural circuits^26–29^. Through their receptors these secreted molecules govern axon growth and synapse formation^30^. Disturbances in the neurotrophin axis in the critical, neonatal period of life could have severe outcomes on future behaviour and nerve system development^31^. In adults stress and GC as an adaptive organism reaction firstly intensify neural transmission and could possibly upregulate nerve growth factor expression but afterwards their action is opposite^32,33^. This twist of GC’s action on organisms comes with prolongation and intensification of GC impact. We are far from detailed and systemic understanding of GC influence on neurotrophins and their receptors expression in the neonatal brain, especially recently discovered (sorcs1-3). These signaling molecules are synthesized in the cells as a proforms, which undergoes proteolytic cleavage to the mature form ^34^. Neurotrophin’s proforms have their own receptor and biological function. Today except classical proneurotrophin receptor *p75ngfr*^34^ it was shown that sorting receptors with vps10 domain *Sorcs2*, *Sorcs3* are also pronerotrophin coreceptors ^35–37^. This sorting receptors are particularly important and crucial for neurons because of its role in other receptors internalization and exposure on membrane^36,38–44^. p75ngfr is crucial for the development of basal forebrain cholinergic neurons, norepinephrine and sympathetic neurons^45^. Complete deletion of *p75ngfr* in knockout mice leads to a significantly elevated number of cholinergic neurons^46^ and abnormal elevation of norepinephrine neurotransmission^47^. Locus Coereleus norepinephrine neurons have massive connections with rostral structures: cerebral cortex, amygdala^48^, hippocampus, especially CA3 gyrus^49^. Main site of p75ngfr’s ligand (*Bdnf*) expression in neonatal forebrain at the same time is the hippocampal CA3 region^50^. In this research we studied *Bdnf* expression in the hippocampus and *p75ngfr* receptor expression in the brainstem after single dexamethasone injection at P2. Another aspect of the study was modulation of elevated glucocorticoids level in neonatal periods with stress during adolescence, which could have paradoxical and opposite results on memory and behaviour at the same time. After chronic mild unpredictable stress during late adolescence period P45-P60 *p75ngfr* and *Sorcs3* expression in the brainstem were studied in comparison with acute stress situation and non-stress animals on the background of neonatal DEX administration.

## Results

### Neurotrophins and their receptors mRNA expression changes after single neonatal dexamethasone injection

**Figure 1:**
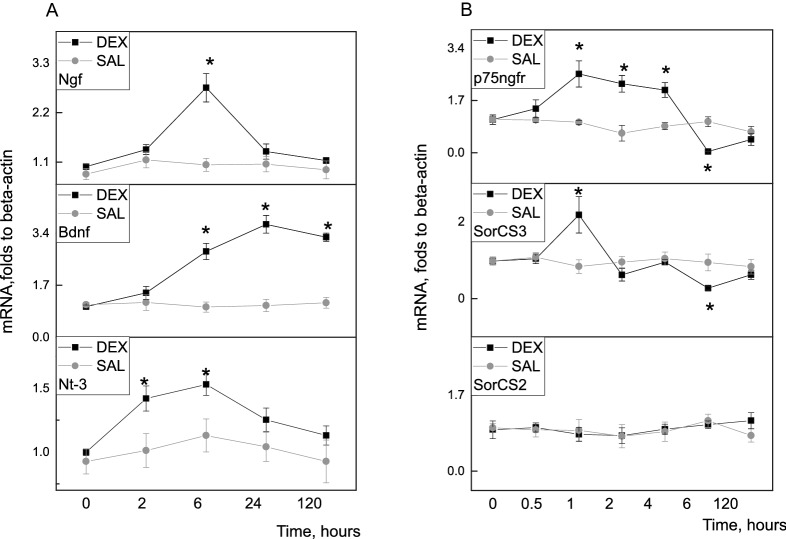
Effects of single DEX injection 0.2 mg/kg to P2 rat pups on neurotrophins in the HPC (**A**) and their receptors in the brainstem (**B**) expression changes. A-*Ngf, Bdnf, Nt-3* timeline expression profile in the HPC after DEX administration B- timeline expression profile of p75ngf, Sorcs2, Sorcs3 in brainstem. ONE WAY ANOVA, *p < 0.05 compared to the control group. Data are presented as M ± SE.

Activity of cortical and hippocampal neurons is known to be involved in depression and behavioural resilience. During early neonatal period neurotrophins are predominantly expressed in this part of the brain, especially *Bdnf*. Disturbances of neurotrophins expression in the hippocampus during early neonatal period by glucocorticoids could have long-lasting programmable effects on behaviour, memory and depression. To answer this question we studied neurotrophins mRNA *Ngf, Bdnf, Nt-3* expression level changes in the hippocampus (HPC) after single subcutaneous dexamethasone injection to P2 rat pups. Timeline changes of mRNA expression are shown on Fig. 1a. We didn't observe any significant changes in mRNA level for all studied genes between SAL treated controls. All *p* > 0.05. For further analysis these groups were merged as control. Notably, *Bdnf *(F(8,50) = 11.129, *p* < 0.05, n = 4–13) and *Nt-3* mRNA level had increased 6 h after DEX administration, but *Bdnf* mRNA level was elevated for longer lime, till 120 h after injection. Interestingly, that (*Ngf* F(8, 58) = 10.693, *p* < 0.05, n = 4–13)and *Nt-3 *(F(8, 54) = 5.2132, *p* < 0.05, n = 4–13) mRNA level had sharp peak at 6 h after DEX injection that was differ from *Bdnf* temporal expression pattern. *p75ngfr and Sorcs3* receptors expression are prominent in the brainstem in the first postnatal week. Brainstem is brain structure where norepinephrine neurons are located. They have massive projections to forebrain structures: cortex and hippocampus^47^. Interestingly, *p75ngfr* expression increased significantly 1 h after DEX administration and decreased significantly at 6 h after DEX (F(6, 39) = 19.008, *p* < 0.05, n = 5–8). Proneurotrophin’s coreceptor *Sorcs3* mRNA level also increased 1 h after DEX and then decreased at 6 h after DEX injection (F(6, 40) = 9.7352, *p* < 0.05, n = 5–8) Fig. 1b. We didn’t observe any changes in *Sorcs2* mRNA expression.

### BDNF and P75NTR protein level after DEX administration

**Figure 2:**
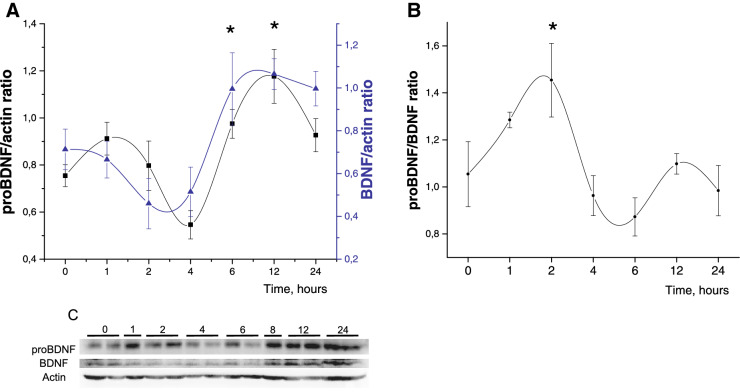
Timeline changes of matBDNF and proBDNF protein level in the HPC after DEX injection to P2 rat pups. (**A**) proBDNF and mature BDNF protein expression level changes (**B**) proBDNF/BDNF ratio changes. ONE WAY ANOVA, *p < 0.05 compared to the control group. Data are presented as M ± SE (**C**) representative western blot image. (original western blot images are accompanied in Supplementary material).

**Figure 3:**
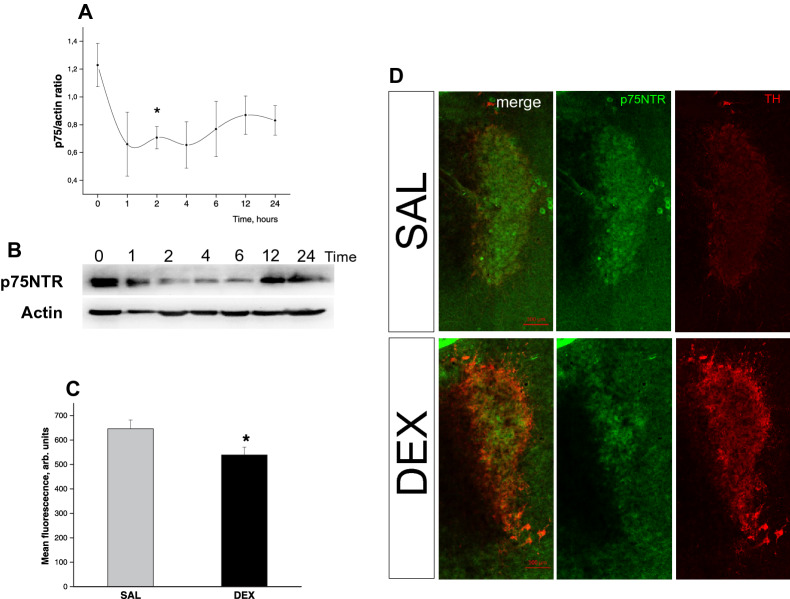
P75NTR protein level changes in the brainstem after single DEX injection 0.2 mg/kg to P2 rat pups. (**A**) p75NTR protein level timeline profile ONE WAY ANOVA, *p < 0.05 compared to the control group, Data are presented as M ± SE (**B**) representative western blot image, (original images are in Supplementary material), (**C**) Immunohistochemistry results.Mean p75NTR fluorescence intensity in norepinephrine LC neurons. ONE WAY ANOVA, *p < 0.05 compared to the control group, Data are presented as M ± SE (**D**) representative micro-photographs of p75NTR (green, Alexa 488) protein level changes in LC norepinephrine neurons(red, Alexa 568) after DEX administration. P75NTR—green (Alexa 488), Tyrosine Hydroxylase (TH)—red (Alexa 568), scale bar—100 mm.

Changes in *Bdnf* mRNA level were accompanied with protein level changes. For the analysis as a control we took samples from different time points of SAL injected rat pups, because we didn’t observe any significant differences between SAL time points on RT-PCR. On the graphs and in the supplement this group is showed as time point “0”. Both BDNF pro- and mature forms levels increased at 6–12 h after DEX administration (F(7, 40) = 5.6414, *p* < 0.05 n = 5–7) proBDNF; F(7, 40) = 4.6248, *p* < 0.05 n = 5–7 BDNF; Fig. 2a (original image blot pictures are accompanied in Supplementary material), but proBDNF/mBDNF ratio had increased only at 2 h after DEX administration F(7, 36) = 2.8863, *p* < 0.05 Fig. 2b,c. p75NTR protein level in the brainstem decreased after DEX injection and didn’t recover 24 h after administration (F(7, 45) = 2.138, *p* = 0.059, n = 5–8); Fig. 3a,b. Double immunohistochemistry of p75NTR with norepinephrine neurons marker tyrosine hydroxylase on brainstem tissue sections Fig. 3c,d showed decreased p75NTR signal in the Locus Coeruleus 6 h after DEX administration F(1, 34) = 5,0448, *p* = 0,03; Fig. 3d. Interestingly, you can notice weak TH signal in SAL treated group and very bright fluorescence in DEX treated group Fig. 3d. This fluorescence signal corresponds to basal TH level. After DEX administration to P2 rat pups we observed elevation of TH activity^51^ and expression^52^ in the brainstem.

### Depressive like behaviour after neonatal dexamethasone administration at P2 and chronic mild unpredictable stress in adolescence

**Figure 4:**
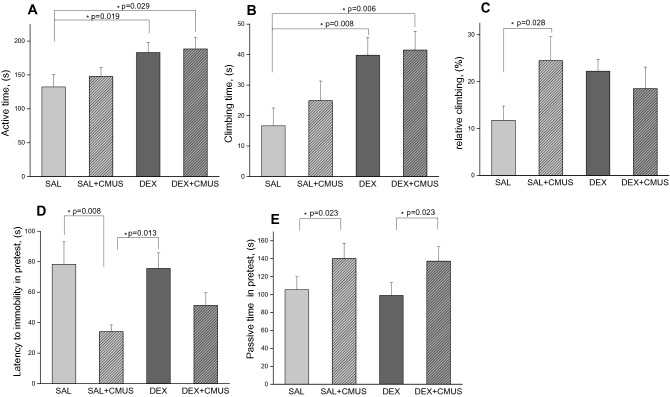
Depressive like behaviour and chronic mild unpredictable stress (CMUS) at late adolescence P45-60 on background of neonatal DEX administration on P2. (**A**) active swimming time in FST, (**B**) Climbing time, (**C**)-Relative climbing time, (**D**) Latency to immobility in the pretest (**E**) Passive time in the pretest session. TWO WAY ANOVA,*p < 0.05, Data are presented as M ± SE.

To evaluate changes in depressive like behaviour caused by single neonatal DEX injection animals at P60 were tested in the forced swimming test. To assess modulatory action of mild stress in adolescence, chronic mild unpredictable stress paradigm (CMUS) were used, starting at P45 till P60. Half of the animals that received either neonatal DEX or SAL injection at P2 were subjected to battery of CMUS stressors. Single neonatal dexamethasone injection leads to changes in depressive-like behaviour at 60th day of life. In the forced swim test (FST) animals that received neonatal DEX injection showed increased active time in FST in both groups with and without chronic stress compare to control with neonatal SAL injection (hormone effect F(1, 39) = 8.185 *p* = 0.007; stress effect F(1, 39) = 0.1 *p* = 0.754; interaction F(1, 39) = 0.425 *p* = 0.518; n = 10–11 Fig. 4a. and decreased passive time. Climbing time in FST was significantly higher after neonatal DEX with and without CMUS compare to control SAL without CMUS (hormone effect F(1, 37)1^¯^0.75 *p* = 0,002; stress effect F(1, 37) = 0.67 *p* = 0.418; interaction F(1, 37) = 0.29 *p* = 0.591; n = 10–11) Fig. 4b. Remarkably, control animals that received neonatal SAL injection and CMUS on 45–60th days of life showed increased relative climbing time in the test Fig. 4c. Latency to immobility wasn’t changed significantly between groups. Chronic mild stress didn’t influence immobility time in the test, but in the pretest session we’ve seen primarily the action of CMUS. In the pretest chronic mild unpredictable stress significantly decreased latency to immobility (hormone effect F(1, 45) = 0.421, *p* = 0.520; stress effect F(1, 45) = 9.487, *p* = 0.04; interaction F(1, 45) = 0.79667, *p* = 0.37684) n = 10–14 Fig. 4d. Passive time in the pretest was affected by CMUS only (hormone effect F(1, 45) = 0.093, *p* = 0.762; stress effect F(1, 45) = 5.537, *p* = 0.023; interaction F(1, 45) = 0.012, *p* = 0.913 n = 10–14) Fig. 4e. Decreased latency and elevated passive time in the pretest session in groups that received CMUS showed that chronic stress was effective. There are numerous studies using CMUS to develop anhedonia in animals and conditions similar to human depression^53^, but a second approach representing depressive-like behaviour as a continuum can also take place, because antidepressants are effective in FST on animals without previous procedures^54,55^.

### *Bdnf* and *Ntf3* mRNA at P60 after single neonatal DEX administration and chronic mild unpredictable stress in the end of adolescence.

**Figure 5:**
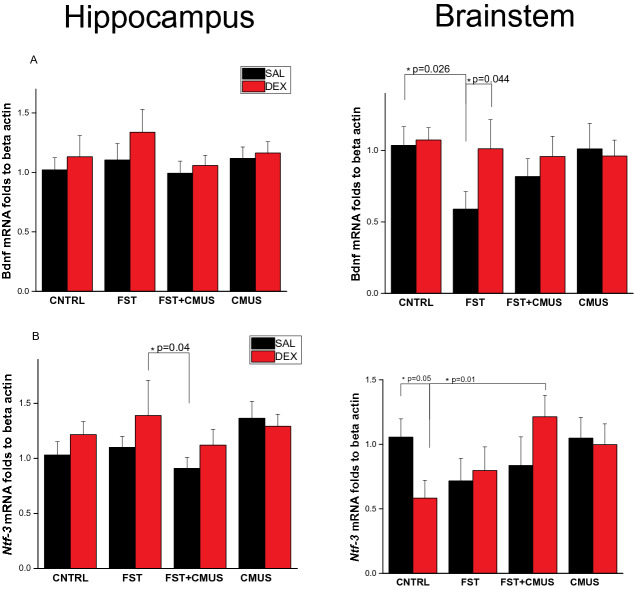
Stress reactive expression of *Bdnf-* (**A**) and *Ntf3-* (**B**) at P60 in the hippocampus and brainstem after neonatal DEX administration and chronic mild stress (CMUS) at the end of adolescence. TWO WAY ANOVA *p < 0.05, Data are presented as M ± SE.

To track long-lasting effects of neonatal DEX administration together with mild chronic stress at the end of adolescence on neurotrophins expression, we’ve conducted qRT-PCR Fig. 5. In the hippocampus we didn’t observe any significant changes in *Bdnf* mRNA level in all experimental conditions Fig. 5a. At the same time in the brainstem we observed two fold decrease in *Bdnf* mRNA level (F(3,38) = 1.26, *p* = 0.026) 2 h after acute stress that was FST itself . Interestingly, that in the group that received neonatal DEX we didn’t observe this *Bdnf* mRNA decrease after FST. Concerning, *Ntf3* we observed similar differences in effects dependent on brain structure. Almost no significant changes in the hippocampus Fig. 4b, and some effects in the brainstem Fig. 4b. Neonatal DEX administration lead to two fold decrease in *Ntf3* mRNA in the brainstem in normal non-stress conditions (F(3,35) = 2.60 *p* = 0.05) Fig. 5b .

### *p75ngfr* and sorting receptors mRNA level in the brainstem and hippocampus after neonatal dexamethasone and chronic mild stress at the end of adolescence in conditions of acute stress

**Figure 6:**
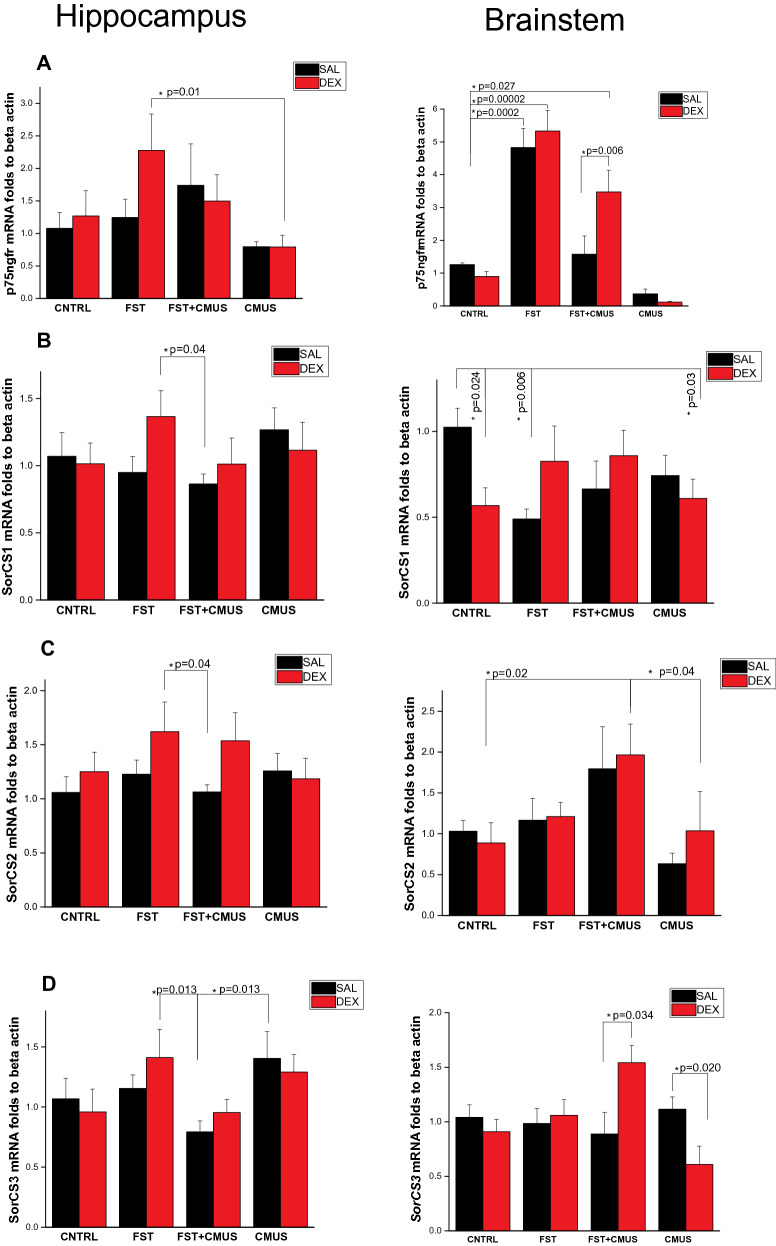
Stress reactive expression of *p75ngfr-* (**A**) and *Sorcs1-* (**B**) *SorCS2*- (**C**), *SorCS3*- (**D**) at P60 in the brainstem after neonatal DEX administration and chronic mild stress (CMUS) at the end of adolescence. TWO WAY ANOVA *p < 0.05, Data are presented as M ± SE.

Changes in proneurotrophin’s receptors mRNA expression level in the brainstem after FST, as well as changes in stress reactive gene expression and consequences of neonatal DEX injection and chronic stress in adolescence (CMUS) were assessed. In the hippocampus we observed 2.5 folds increase in p75ngfr mRNA after FST only in the group that received neonatal DEX administration (F(3, 38) = 2.49, *p* = 0.01 compare to CMUS group), but not in the respective SAL injected control group Fig. 6a. Notably, *p75ngfr* expression increased 5 folds in the brainstem 2 h after FST (hormone effect F(3, 31) = 1.73, *p* = 0.199; stress effect F(3, 31) = 39.64, *p* < 0.05; interaction F(3, 31) = 2.46, *p* = 0.081, n = 4–6) Fig. 6a. Significant differences in *p75ngfr* expression between groups with neonatal DEX and SAL injection were seen when rats received CMUS and after that FST. CMUS decreased *p75ngfr* mRNA level in the group that received neonatal SAL injection to the control level, but couldn’t decrease to control level in the group that received neonatal DEX injection; Fig. 6a. We didn’t observe much significant differences in SorCS1 expression in the hippocampus Fig. 6b. In the brainstem we’ve seen two fold decrease in SorCS1 mRNA level in the CNTRL group that received neonatal DEX (F(3, 36) = 3.48, *p* = 0.024 Fig. 6b). After FST SorCS1 mRNA level was decreased the same rate Fig. 6b, but not in the group that received neonatal DEX Fig. 6b. In the hippocampus we didn’t see so much significant differences in SorCS2 expression between studied groups Fig. 6c. In the brainstem we’ve seen two fold increase in SorCS2 mRNA level in the group CMUS + FST (F(3, 37) = 4.08, *p* = 0.013). Interestingly, that FST itself didn’t change SorCS2 expression in the brainstem. We observed slightly ~ 1.5folds elevation of SorCS3 mRNA level in the hippocampus after chronic mild stress (F(3, 39) = 3.75, *p* = 0.018) and slightly decrease in the group that received both CMUS + FST Fig. 5d. FST didn’t influence on *sorcs3* expression in the brainstem (hormone effect F(3, 37) = 0.049, *p* = 0.825; stress effect (F(3, 37) = 1.977, *p* = 0.134; interaction F(3, 37) = 5.372 *p* = 0.004, n = 4–6) Fig. 6b. CMUS together with FST increased *sorcs3* mRNA in the animals that received neonatal DEX 6b. In the animals that come through CMUS *sorcs3* expression decreased in the group with neonatal DEX injection compared to SAL control Fig. 6b.

## Discussion

Single neonatal dexamethasone administration leads to *Bdnf, Nt-3 and Ngf* mRNA expression elevation in the hippocampus 6 h after injection. This rising could be explained by acute glutamatergic neurotransmission intensification by glucocorticoids after injection^4^. Observed raise of mRNA level was postponed to 6 h after injection, but neuron excitation and glutamate burst after glucocorticoid injection were observed in 30 min after administration^4,23,56^. Besides changes in mRNA level similar changes were observed on BDNF protein level. proBDNF and mature BDNF protein level increased 6 h after DEX injection. proBDNF/matBDNF ratio elevated only 2 h after DEX injection, could be explained by small matBDNF level decline. Neither matBDNF, nor proBDNF level didn’t change significantly in 2 h after DEX injection. Growth factors expression changes in HPC were accompanied with *p75ngfr* and *Sorcs3* receptors expression changes in brainstem–brain structure where catecholamine neurons are located^48^. Lesions and disturbances in catecholamine neurotransmission in the neonatal period lead to behavioural abnormalities in adult age^36,51,57–60^. Norepinephrine neurons project to different rostral areas, including hippocampus^48,49^, where it has a neuromodulatory role and participates in attention acquisition, learning and memory^61–63^. Norepinephrine transmission abnormalities result in maladaptive behaviours including those expressed in addiction^64^, ADHD^36,38^, schizophrenia, depression and post-traumatic stress disorder ^65^. Interestingly, that dexamethasone injection firstly increased *p75ngfr* and *Sorcs3* mRNA level and afterwards decreased at 6 h after injection. No effect on *Sorcs2* mRNA expression level in the brainstem was observed. Decline in proneurotrophin receptors mRNA level (*p75ngfr* and *Sorcs3*) could be explained by presence of predicted negative nGRE elements in their promoters^16^. Glucocorticoid receptors occupy responsive elements in the genome within 30 min after injection^66^, but for the genes repression some time after receptor’s action on the genome should pass to decrease mRNA that were already present in the cell^16^. For *p75ngfr* and *Sorcs3* 4–6 h is needed to decrease mRNA level. Similar changes were observed on p75NTR protein level in the brainstem and locus coerelus norepinephrine neurons, according to immunohistochemistry. After single DEX injection on 2nd day of the life we observed increased expression of *Bdnf, Nt-3, Ngf* in the HPC and decline in proneurotrophin receptor’s expression in brainstem and LC, that could lead to increased norepinephrine neurons axonal growth and norepinephrine transmission elaboration^51,52,67^. Rats that received single neonatal DEX administration showed increased active time in FST. Interestingly, climbing in FST, that is affected by norepinephrine antidepressants^54,55^ were also significantly higher after neonatal DEX. These changes in depressive-like behaviour could reflect norepinephrine transmission intensification after neonatal DEX^51,52,67^. Influence of neonatal injection in tapering doses was intensively studied. It increased anxiety and depressive-like behaviour in adult rats^68,69^. Effects of tapering, prolonged and high dose (1–5 mg/kg) DEX injection are incomparable with single low dose (0.1–0.2 mg/kg) administration. Besides the effects on depressive behaviour neonatal triple dexamethasone injection leads to learning and memory impairment and cognitive deficit^20,70–72^. Observed by us behavioural outcome is similar to behavioural phenotype of mice with forebrain MR overexpression^73,74^. Neonatal DEX leads to long-lasting GR’s expression reduction^75^ and epigenetic^76–78^ changes of different genes^73,79^ and brain structures^80^. Because of it MR signaling could prevail and animals that received neonatal DEX injection repeat MR overexpression phenotype. Epigenetic and DNA methylation changes after DEX administration in critical periods could possibly deregulate brain GR expression and influence on stress reactivity in this way^81,82^. In this work we showed that altered GR signaling changed *Bdnf* and neurotrophins expression in the hippocampus in the neonatal period. Changes of GR regulation could be observed not only after hormones or ligands administration. Acute and chronic stress strongly influence GR expression and signaling. The second critical time point of development is adolescence and transition time period directly preceding adulthood. Chronic stress during adolescence perturb different types of behaviour in adults: reproduction behaviour, cognition, and the ability to respond to adversity typically^83^. It modulates adult stress response in sex specific manner and change differently hippocampal transcriptome and impact global DNA methylation^84^. Remarkably, that chronic stress during adolescence impairs and improves learning and memory at the same time in adults^83^. Adolescent-stressed rats exhibited enhanced reversal learning, an indicator of behavioural flexibility, but showed no change in associative learning and reference memory abilities. Even more interesting is the fact that working memory in these rats, which in humans is thought to underpin reasoning, mathematical skills, may be enhanced, but after novel condition disturbances it decreases. Different types of stressors (predictable, unpredictable and restraint) that rats received during the end of adolescence had different effects on fear memory extinction via *Bdnf* expression and methylation changes in infralimbic cortex and Bdnf dependent ERK1/2 kinase^25^. Although our work was done only on males, and sex differences were out of focus of this study, but women’s more likely than men to develop stress related disorders and are more vulnerable to stress in adolescence. Molecularly sex specific differences in the reaction to the stress in adolescence and changes in the hippocampal transcriptome in adulthood could be determined by estrogen receptor alpha (ESR1) signaling and glucocorticoids–estrogen crosstalk^84^.

Remarkably that most of the studied genes were changed in the brainstem at P60 and at the lesser extent in the hippocampus Figs. 5 and 6. Interestingly that, according to our behaviour data we’ve seen decrease in *Bdnf* mRNA in the brainstem after FST. In the group with neonatal DEX injection it reversed to the unstressed control. This fact could partly explain decrease in depressive-like behaviour after single neonatal DEX injection observed by us. In the unstressed group expression only of Ntf3 and SorCS1 in the brainstem was decreased significantly between SAL and neonatal DEX groups. In rats Ntf3 is expressed in norepinephrine LC neurons^85^. Imipramine and desipramine treatment decreases Ntf3 mRNA in LC neurons^85^. Among receptors only p75ngfr, SorCS1 and SorCS3 had different stress-reactive expression in the brainstem dependent on neonatal DEX administration. Remarkably, that this effect is also brain structure dependent. It could be explained by different effects of neonatal DEX on GR posttranslational modifications and epigenetics in brainstem and hippocampus. At P60 alteration of *Sorcs3* and *p75ngfr* receptors expression in groups with neonatal DEX administration was significantly different from control only after CMUS or CMUS and acute stress, which was in our case FST itself. CMUS influence strongly on GR expression^86^ and GR, hsp90 posttranslational modification^87,88^. Predicted nGRE in the *Sorcs3* and *p75ngf* promoters could possibly regulate expression of these genes after chronic stress and because of it their expression level could be differ in the groups with neonatal DEX. *p75ngfr* expression increased fivefold after FST i.e. acute stress. The Receptor's expression is controlled not only by glucocorticoids, but also by different factors, including neuronal activity. Arousal that is observed in acute stress leads to extensive neurotransmission outburst^89^. Free oxygen species that rise after acute stress cause neuronal oxidative stress. *p75ngfr* expression is elevated dramatically after acute stress. *p75ngfr* is implicated in axonal degeneration^90^ and apoptosis induced by oxidative stress via ligand independent mechanism^91^. This fact could possibly explain neuron cell death observed after acute stress. But simplifying the *p75ngfr* role to death receptor is mistakable, because it could be also prosurvival. On neuronal cell membrane it can dimerize with tyrosine kinase receptors and execute this function^92^. In the PC12 cell line with norepinephrine phenotype *p75ngfr* knockdown lead to the cell death as well^93^. Interestingly, that in PC12 cells only mature NGF and proNGF-A could promote neurotrophic effect through *p75ngfr*^94^. In overall, *p75ngfr* play crucial role in balancing prosurvival-versus-death balance in neurons^95^. Notably, *p75ngfr* play its role in orchestrating survival balance together with vps10 sorting receptors, including *Sorcs3*^35,36^. Sorting receptors take part in other transporters and receptors internalization and exposure on neuronal membrane. In summary, our results demonstrate that single neonatal DEX administration acutely changed neurotrophins expression in the HPC and *p75ngfr* and *sorcs3* in the brainstem and LC norepinephrine neurons. At P60 single neonatal DEX injection leads to decrease in depressive-like behaviour, observed in FST, independently from chronic stress. Neonatal DEX administration decreased *Ntf3* and *SorCS1* expression in the brainstem. Also *Bdnf* mRNA level in the brainstem of these animals didn’t decrease after FST. Chronic mild unpredictable stress at the end of adolescence changed *p75ngfr* and *SorCS3* expression in the brainstem of the animals that received single neonatal DEX administration 0.2 mg/kg.

## Methods

### Animals and experimental design

The study was carried out in compliance with the ARRIVE guideline. All animal procedures were approved by the Institute’s Cytology and Genetics SB RAS Animal Care and Use Committee and conducted in compliance with the European Communities Council Directive 63/2010/EU. Pregnant Wistar rats were individually housed (22°–24°, natural light) with free access to food. The day of birth was considered as P0. Litters were restricted to 8 male pups per litter. If there were less than 8 males in the litter the rest were cross fostered from other dams. In the same litter were animals from all experimental groups. Dexamethasone phosphate (KRKA) 0.2 mg/kg or saline were injected subcutaneously on the 2nd day of the life P2. After indicated time points for expression studies animals were sacrificed, brain structures were isolated and snap freezed in liquid nitrogen. Pups were separated from mother on the 24th day of the life and housed 8 sibs in the cage.

### Chronic mild unpredictable stress (CMUS) paradigm

Starting from the 45th day of life half animals from each group were exposed to unpredictable mild chronic stress. Our paradigm consist of battery of seven stressors, that were randomized during 2 weeks: (1) 1 h at the 45° angle tilted cage, (2) 10 min warm air steam from hairdryer, (3) 1 h immobilization in the cylinder, (4) 10 min swimming in the 30 × 40 cm water tank, (5) 24-h food deprivation, (6) light time extension to 24-h, (7) 24 h at the cage with 16 new neighbors each time. Each day every cage of animals received a new stressor from the battery, that didn’t coincide with previous day test’s. CMUS paradigm took place at P45-P60. All animal procedures were done till 15 h.

### Behaviour testing

Depressive-like behaviour was characterized using forced swim test. The day before test animals had 15 min pretest in a glass 30 × 60 cm swimming cylinder filled with water t = 25 °C. Following day 5 min. test session was done using the same setup. Pretest and test sessions were recorded on camera for further quantification and analysis. Total immobility time, active time, latency to immobility and climbing time were counted by three independent observers. For the mRNA expression analysis brains were taken 2 h after FST.

### Real-time RT-qPCR analysis

Total cellular RNA was isolated using a single-step acidic phenol extraction as previously described^4^. 1 µg total RNA was reverse transcribed with the 100U MMLV Reverse Transcriptase (Sibenzyme), 1 mM dNTP, 2 mM DTT, 2 µM OligodT primer (Evrogen) and standard thermocycler temperature conditions for MMLV. All real-time PCR reactions were performed using the ABI ViiA7 system (Thermo) and standard cycle. Amplifications were done using the real time PCR Master Mix qPCRmix-HS + LowROX (Evrogen) and primers and Taqman probes from the Supplementary Table 1.

### Western blot

Western blot was conducted as described previously^96^. Brain tissue was homogenized in a lysis buffer (150 mM NaCl, 50 mM Tris, 1% Triton X-100, protease inhibitors: 2 mM PMSF and 2 µg/mL leupeptin, pepstatin, and aprotinin). After centrifugation (14,000 g, 4°C, 15 min), the protein concentration was measured by the Lowry assay. Supernatant containing 50 µg of protein was denatured in a 1 × Laemli buffer (50 mM Tris–HCl (pH 6.8), 10% glycerin, 100 mM *β*-mercaptoethanol, 1% SDS, 0.002% bromophenol blue; 5 min at 95°C). Afterwards samples were separated by SDS electrophoresis (Mini-Protean Cell chamber, Bio-Rad Laboratories, United States) in 12% polyacrylamide gel (size 84 × 80 × 1 mm). Electrophoresis was performed in an electrophoretic buffer (25 mM Tris, 192 mM glycine, 0.1% SDS, pH 8.3) at a voltage of 120 V until the samples entered the separating gel, then at 180 V. After electrophoresis, the proteins were transferred to a 0.45 µm nitrocellulose membrane (Bio- Rad Laboratories, United States) in a transfer buffer (47.9 mM Tris, 38.6 mM glycine, 20% methanol, pH8.3) at a constant voltage of 70 V for 60 min (Trans-Blot, Bio-Rad Laboratories, United States). Membranes were incubated overnight at 4°C on rotary platform with following antibodies: (BDNF 35928.11, mouse monoclonal, dilution 1:500, Milliore, United States; p75NTR (D4B3), rabbit monoclonal, dilution 1 : 500, Cell Signaling, United States; *β*-actin, sc-1616, rabbit polyclonal, dilution 1:20,000, Santa Cruz Biotechnology, United States) and secondary (goat anti-rabbit IgG antibody, dilution 1:1000 Bio-Rad Laboratories, United States). The chemiluminescence signal was amplified using a special kit (SuperSignal West Femto Maximum Sensitivity Substrate, Lifetechnologies) for 1 min. The staining intensity of the bands corresponding to the analyzed proteins was assessed by scanning the membranes (Chemidoc Touch Imaging System, Bio-Rad Laboratories, United States) followed by digital densitometry (Scion Image 4.0.3.2 software Scion Corporation, United States).

### Immunohistochemistry

P2 rat pups 6 h after subcutaneous DEX injection were deeply anesthetized with avertin and transcardially perfused with 1xPBS and subsequently with 4% PFA. Brains were postfixed for 4 h in 4% PFA and cryoprotected in 30% sucrose overnight. 25 µm frozen tissue sections of brainstem in LC region were prepared with MICROM350 cryostat according to neonatal rat brain atlas (https://www.ial-developmental-neurobiology.com/en/publications/collection-of-atlases-of-the-rat-brain-in-stereotaxic-coordinates). Sections were mounted on Super Frost Plus slides (ThermoFisher). Sections were washed in 1xPBST 0.2% Triton X-100. Nonspecific bindings were blocked with 5% donkey serum in 1xPBST for 1 h at RT. Subsequently section were incubated overnight at 4°C with primary antibodies: AB1542 sheep anti tyrosine hydroxylase, Millipore 1:300, 4201 monoclonal rabbit anti p75NTR (D4B3), Cell Signaling 1:300 diluted with blocking buffer. Then sections were washed 3 times for 10 min in 1xPBST. Subsequently sections were incubated with F(ab)2-donkey anti-sheep Cy-3, Jackson Immunoresearch and F(ab)2-donkey anti rabbit Alexa 488, Jackson Immunoresearch secondary antibodies. Afterwards slides were washed 3 times for 10 min in 1xPBST and subsequently were mounted coverslips with MOWIOL containing nuclear counterstain DAPI. Sections were analyzed by confocal laser-scanning microscopy (Zeiss LSM 780) at the Microscopic Centre of the Institute of Cytology and Genetics, Novosibirsk, Russia. The following channel settings were used for CLSM imaging: DAPI—405 nm, FITC and Alexa 488–488 nm, Cy3 with 561 nm laser lines. Panoramic images of the brainstem in the LC region were created using a tile scan function (Plan-Apochromat 20x/0.8 M27 objective and 200 µm pinhole). There were 4 animals in each group. 5 confocal images were acquired from each animal. Mean fluorescence levels in the LC region were measured in ZEN software (Carl Zeiss).

### Statistics

Statistical analyses were performed using STATISTICA software. For timeline gene expression and immunohistochemistry ONE WAY ANOVA analysis were used. For the behavioural analysis, p75ngfr and sorcs3 expression analysis after CMUS paradigm TWO WAY ANOVA were used. All significant values were further analyzed using Fisher LSD post-hoc analysis.

## Supplementary Information

Supplementary Information

## Data Availability

The data that support the findings of this study are available from the corresponding authors upon reasonable request.
