# Supplementary material for: Single neonatal dexamethasone administration has long-lasting outcome on depressive-like behaviour, Bdnf, Nt-3, p75ngfr and sorting receptors (SorCS1-3) stress reactive expression
Source: Sci Rep. 2021 Apr 14;11:8092. doi: 10.1038/s41598-021-87652-7 (PMC8046778; doi:10.1038/s41598-021-87652-7)
Supplement: Supplementary file 1 — Supplementary Information [file 41598_2021_87652_MOESM1_ESM.pdf]

Title: Single neonatal dexamethasone administration has long-lasting outcome on depressive-like behaviour Bdnf, Nt-3, p75ngfr and sorting receptors (SorCS1-3) stress reactive expression.

Lanshakov D. A.[1,*], Sukhareva E. V.[2,3], Bulygina V. V.[2], Bannova A. V.[2], Shaburova E.V. [2,3], Kalinina T. S.[2,3]

[1]Laboratory of Postgenomics Neurobiology, Institute of Cytology and Genetics, Russian Academy of Science, Novosibirsk, 630090, Russian Federation.
[2]Functional Neurogenomics Laboratory, Institute of Cytology and Genetics, Russian Academy of Science,
Novosibirsk, 630090,
Russian Federation.
[3]Department of Natural Sciences, Novosibirsk State University, Novosibirsk, 630090, Russian Federation.
[*]corresponding to dmitriylanshakov@gmail.com, lanshakov@bionet.nsc.ru

Supplementary Material

Table 1. List of oligos and taqman assays used in the study.

gene	sequence/cat. no
b-actin	Rn00667869_m1(Thermo)
bdnf	Rn02531967_s1(Thermo)
p75ngfr	Rn00561634_m1(Thermo)
ntf3	Rn00579280_m1(Thermo)
ngf	Rn01533872_m1(Thermo)
sorcs3F	TGGGACTTCGAATGTGACTATG
sorcs3R	GTACCCAGTGCTGTTAAGGTAG
sorcs3prb	/6-FAM/TGAGAGACATGGAGAGAGCCAGTGT/BHQ-1/
sorcs2F	CTACGAGAGTCCTGGCATCTA
sorcs2R	GGAGAGTTGACCTGGACAAAG
sorcs2prb	/6-FAM/CGTGTGTCTGTCAGGGCTGAGAAC/BHQ-1/
sorcs1F	ATGGGAGCCAAGAGGATCTA
sorcs1R	CGTAGCCATAGTCACAGTCAAA
sorcs1prb	/6-FAM/TATGCAAGGGACGTATGCAGGAGC/BHQ-1/

.

2012-2014 Dex dynamic 1,2,4,6,8,12,24h CR BDNF

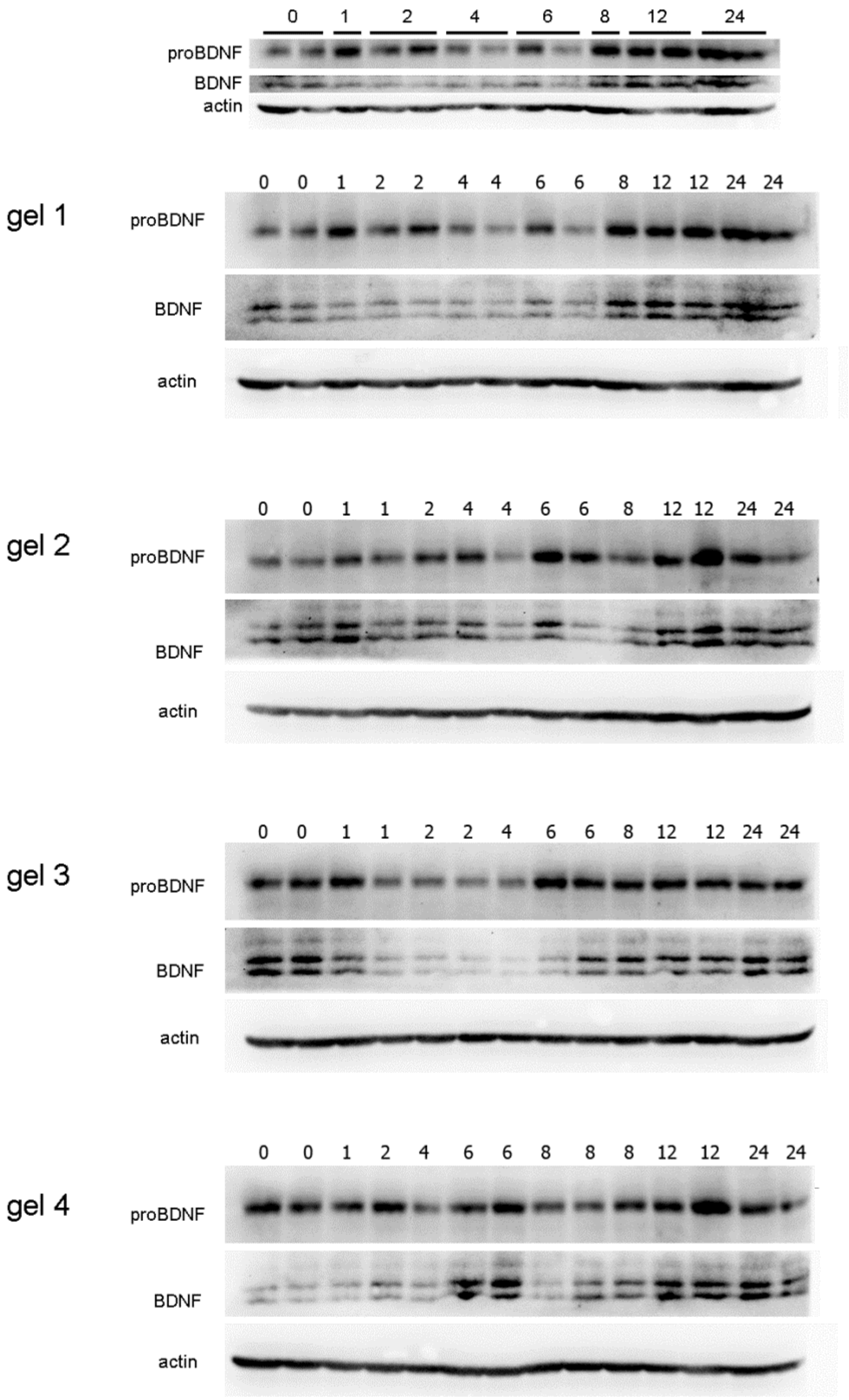

Actin for mature BDNF and proBDNF

gel 1

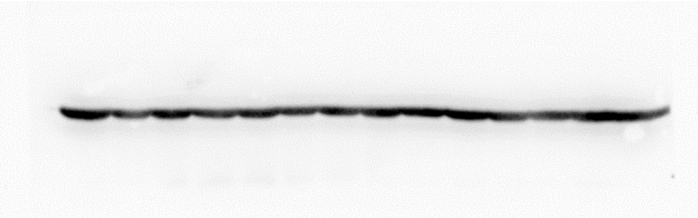

gel 2

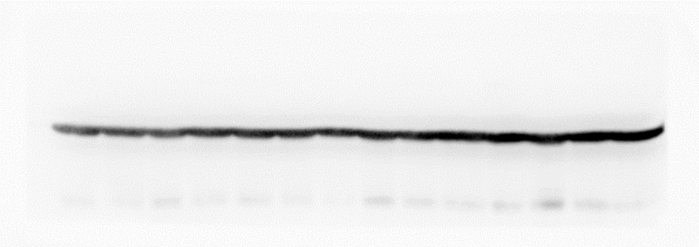

gel 3

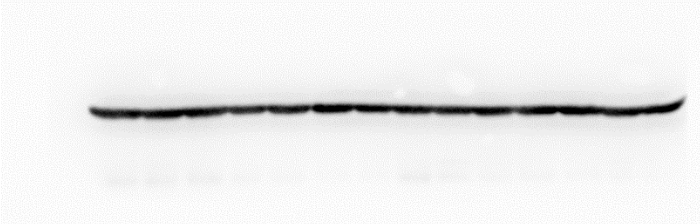

gel 4

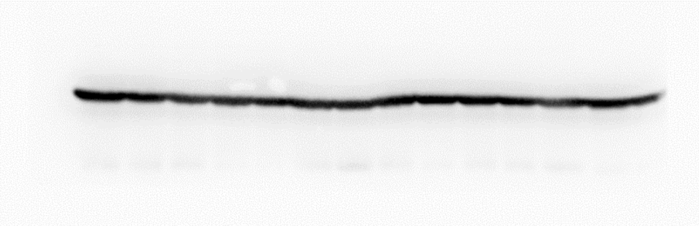

proBDNF

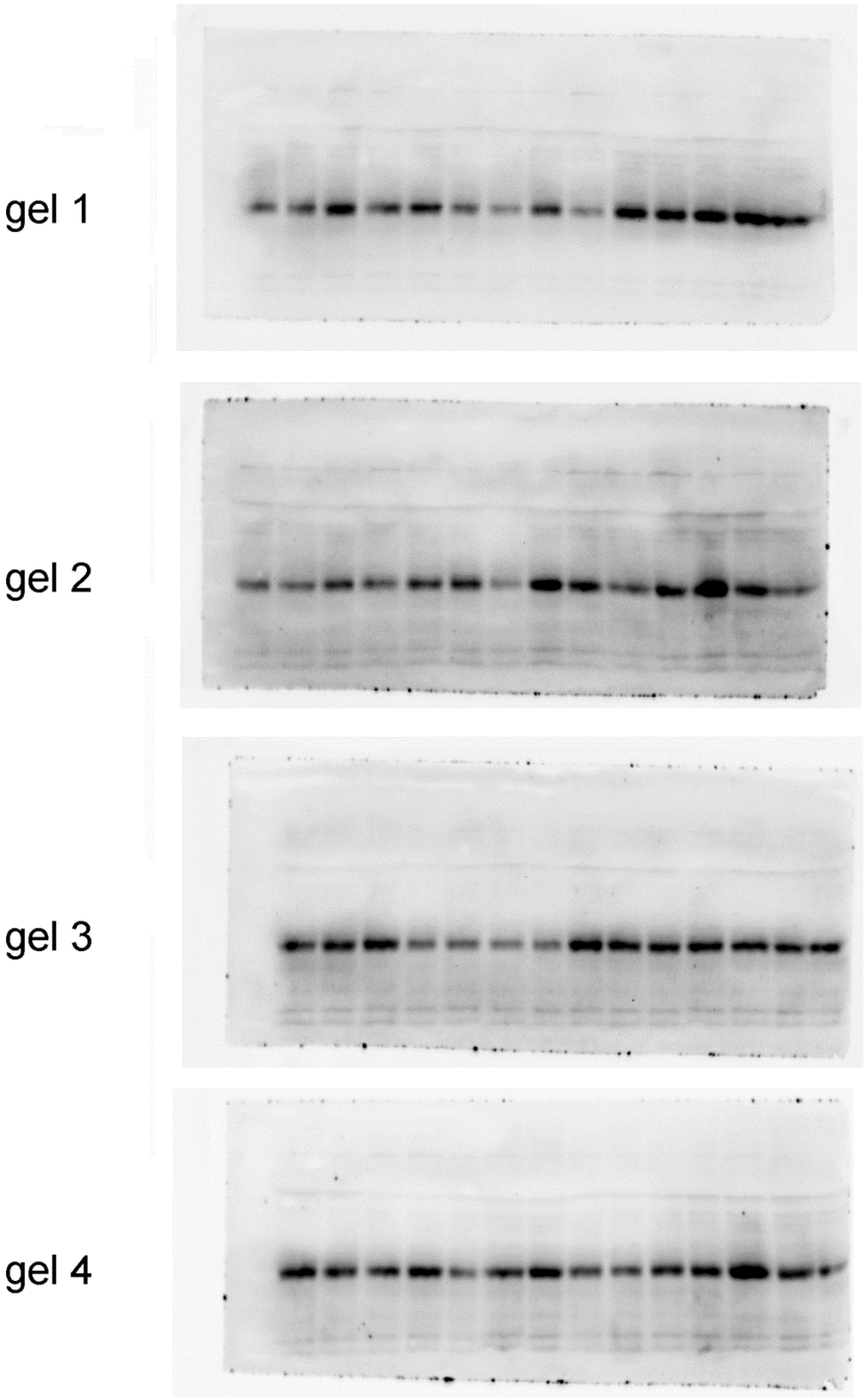

mature BDNF

gel 1

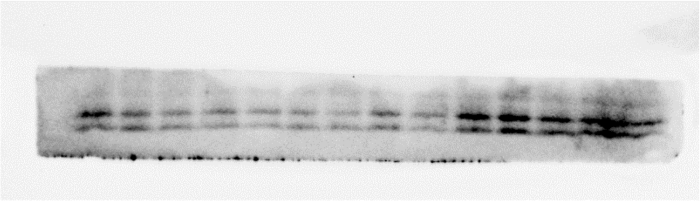

gel 2

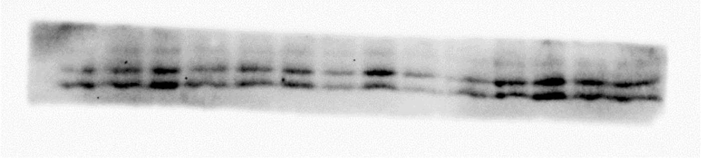

gel 3

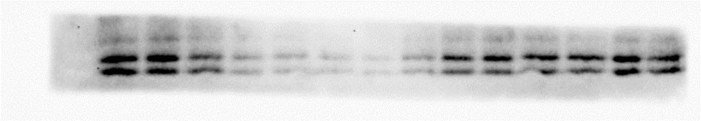

gel 4

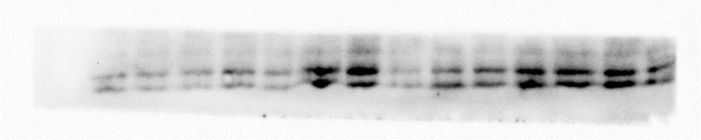

2012-2014 Dex dynamic 1,2,4,6,8,12,24h ST

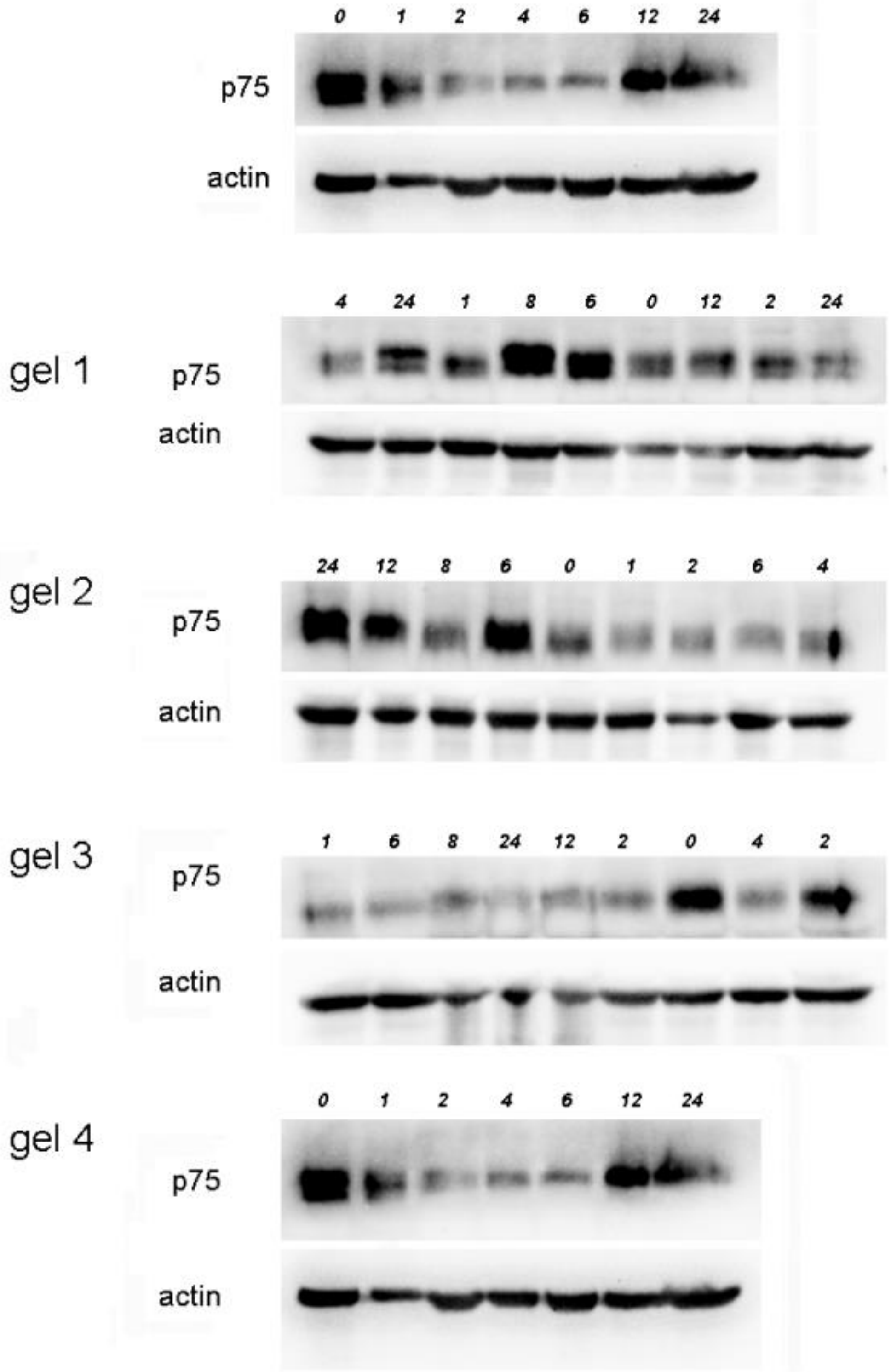

Actin for p75

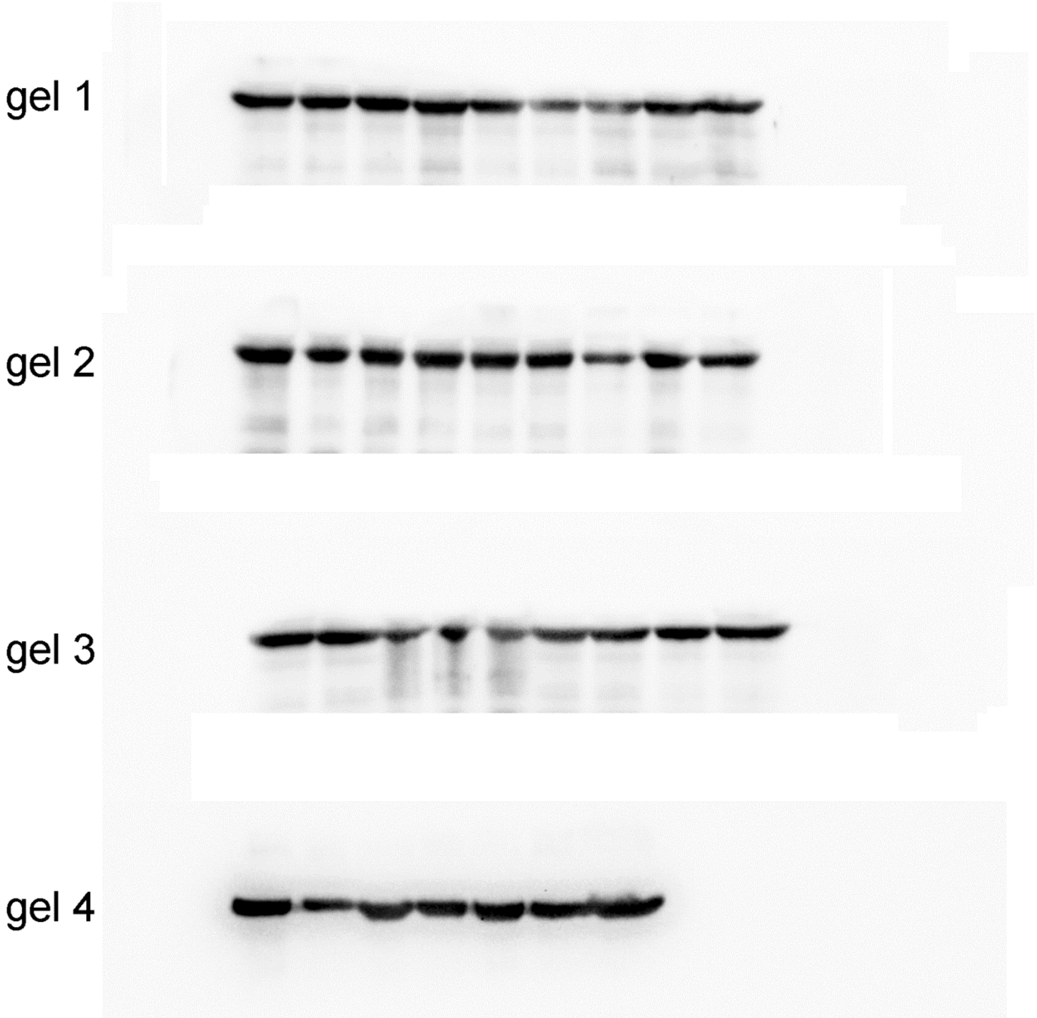

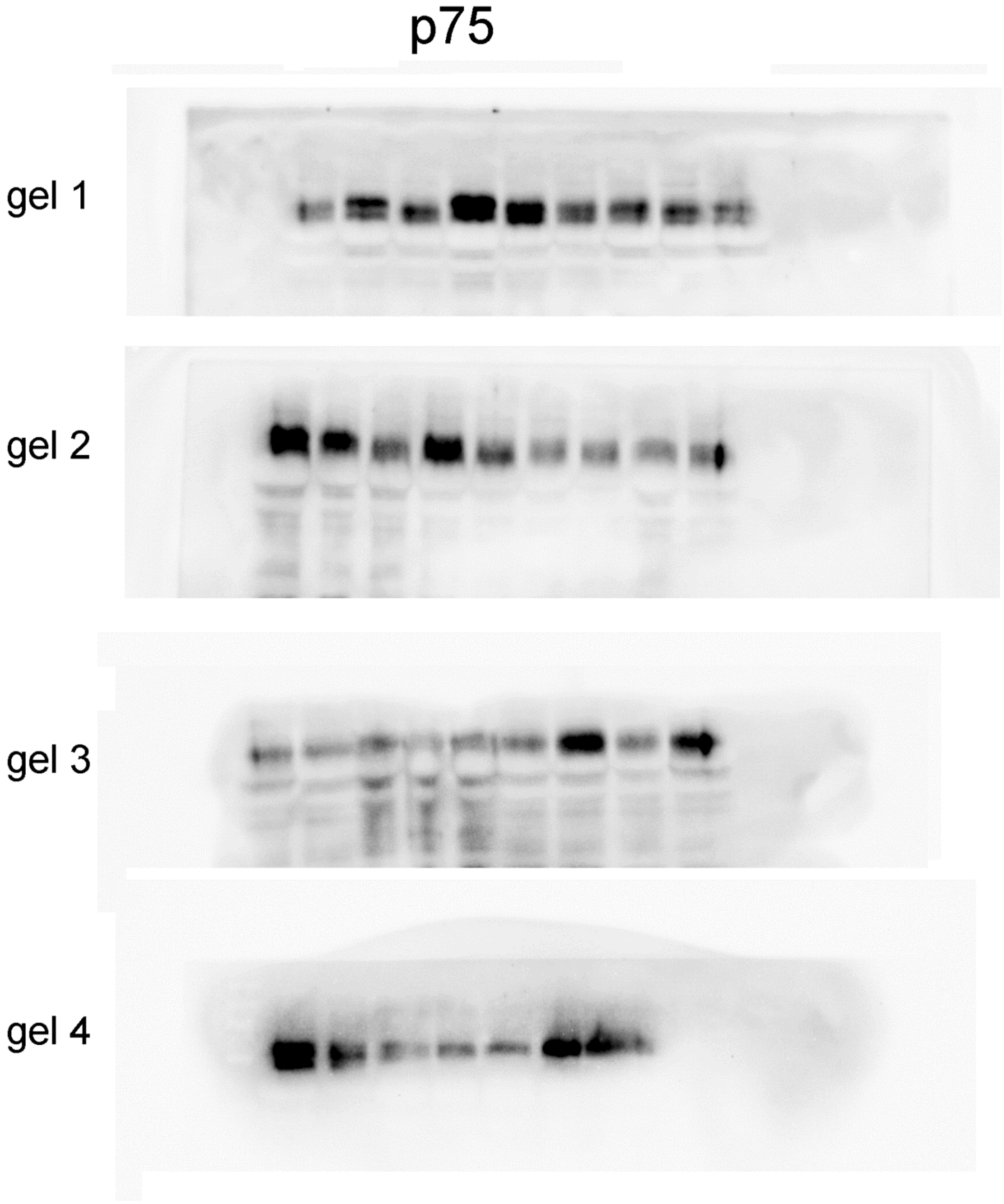
p75
gel 1
gel 2
gel 3
gel 4
